# Sinusoidal Wave Estimation Using Photogrammetry and Short Video Sequences

**DOI:** 10.3390/s151229828

**Published:** 2015-12-05

**Authors:** Ewelina Rupnik, Josef Jansa, Norbert Pfeifer

**Affiliations:** Department of Geodesy and Geoinformation, Technische Universität Wien, Gusshausstrasse 27-29, Vienna 1040, Austria; Josef.Jansa@geo.tuwien.ac.at (J.J.); Norbert.Pfeifer@geo.tuwien.ac.at (N.P.)

**Keywords:** photogrammetry, video, 3D modeling, bundle adjustment, specular, non-rigid, dynamic, water, sinusoidal wave

## Abstract

The objective of the work is to model the shape of the sinusoidal shape of regular water waves generated in a laboratory flume. The waves are traveling in time and render a smooth surface, with no white caps or foam. Two methods are proposed, treating the water as a diffuse and specular surface, respectively. In either case, the water is presumed to take the shape of a traveling sine wave, reducing the task of the 3D reconstruction to resolve the wave parameters. The first conceived method performs the modeling part purely in 3D space. Having triangulated the points in a separate phase via bundle adjustment, a sine wave is fitted into the data in a least squares manner. The second method presents a more complete approach for the entire calculation workflow beginning in the image space. The water is perceived as a specular surface, and the traveling specularities are the only observations visible to the cameras, observations that are notably single image. The depth ambiguity is removed given additional constraints encoded within the law of reflection and the modeled parametric surface. The observation and constraint equations compose a single system of equations that is solved with the method of least squares adjustment. The devised approaches are validated against the data coming from a capacitive level sensor and on physical targets floating on the surface. The outcomes agree to a high degree.

## 1. Introduction

Attempts to characterize the water surface with optical methods date back to the beginning of the 20th century [1,2]. The interest in a quantitative description of the surface with light came from the field of oceanography and the use of photography to map the coastlines. This prompted further applications, namely the use of photography to quantify ocean waves and to exploit these parameters in, e.g., shipbuilding, to engineer structures of the appropriate strength [3,4,5].

The same drivers disseminated optical methods among other applications, in river engineering and the oceanographic domain. Understanding river flow allows for a better riverbed management and mitigation of floods through combined fluid dynamics modeling and experimental testing. Additionally, the knowledge of the dispersive processes gives an insight into the way pollution and sediments are transported [6,7,8,9].

In the coastal zones, optical methods became a good alternative to *in situ* measurements, which require substantial logistical commitments and offer low spatial, as well as temporal resolution. The dynamics of the water, hence the energy it carries, influences the nearshore morphology, which is of significance for both coastal communities and marine infrastructure, e.g., wharfs and mooring systems [10,11,12,13].

Last, but not least, the roughness of the ocean’s surface is meaningful from the viewpoint of exchange processes with the atmosphere. The oceans, accounting for two-thirds of the Earth’s surface, contain high concentrations of carbon dioxide and thereby influence the global climate system through the evaporation and absorption phenomena. The optically-measured wave slope can be used to parametrize the relationship between the surface roughness and gas transfers, as well as the wind speed and direction [14,15].

Other recent and operational approaches to ocean surface observations are radar altimetry [16] and GNSS reflectometry [17].

### 1.1. Contributions

**Figure 1:**
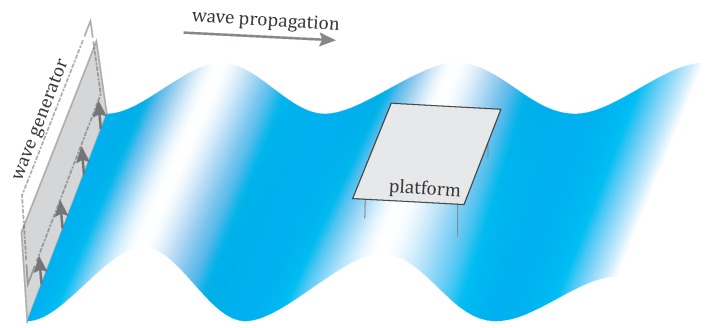
A simplistic view of the model basin. The platform is placed at a distance from the wave generator (on the left) and parallel to the wave propagation direction.

The objective of this work was to observe the motion of a floating platform, simultaneously providing information on the forces generating the motion, *i.e.*, the shape of the water waves (*cf.* Figure 1). The waves were generated with a linear mechanical arm, occupying the entire width of the basin. The wave propagation followed a single direction along the basin’s longer axis. As such, the arm moved at equal time intervals producing sinusoidal waves at various, but constant frequencies (*f*), periods (*ω*) and wavelengths (*λ*); *cf.* Figure 1. The water was rendered a mirror-like surface, while the testing facility was in large part occupied by a model basin and offered little space around the measurement volume. Hence, the challenges were split between the uncommon surface characteristics, the workplace constraints, as well as the non-professional, budget-conscious imaging equipment employed for the timely observations. The contributions of this article include: (i) a concept for exploiting specular reflections of light sources to model the water surface, (ii) a method for measuring and modeling specular reflections of light sources in an image and 3D reconstruction of the dynamic wave shape from it, plus; (iii) an evaluation of the suggested method using the capacitive level sensor and physical targets freely floating on the surface’s top.

Two methods are proposed, treating the water as a diffuse and as a specular surface, respectively. In either case, the water is presumed to take the shape of a traveling sine wave, reducing the task of the 3D reconstruction to resolve the wave parameters. This was accomplished in a two-fold manner, by observing: (i) a few physical targets floating on the top of the surface (Method 1 in Section 3.1), and (ii) the apparent motion of specular reflections coming from points of known coordinates (Method 2 in Section 3.2). In either case, the transfer from point-based measurements to a surface was possible thanks to the prior knowledge of the wave excitation.

The determination of the platform’s motion is out of scope of this publication and was presented in [18].

### 1.2. Related Works

One can distinguish between three groups of measurement methods, each exploiting different characteristics of the water medium. The water surface can be regarded as (1) diffuse-like (quasi-Lambertian), (2) mirror-like (specular reflection) or else (3) as a transparent medium (refraction). When the wavelength of the incident radiation is smaller or equal to that of the surface roughness, the surface will appear as diffuse. On the other hand, smooth surfaces will produce specular reflections, and their appearance will be highly dependent on the position of the observer, as well as the radiation source [19]. The majority of approaches utilize passive sensors in reconstructing the surface shape. Active illumination techniques applied to highly reflecting surfaces produce systematic errors in depth estimation. There are few recorded attempts to adapt the scanning techniques to deal with the specularity effects, in particular by space-time analyses of the signal and filtering procedures [20].

#### 1.2.1. Water as a Diffuse Surface

In practice, there are two conditions when the water is rendered diffuse-like: first, when the surface is disturbed with small waves (e.g., rough sea in the coastal zone); second, when artificial targeting is employed. The most common targeting techniques use physical material, like powder, Styropor, oil or optical projections in the forms of a laser sheet, a grid of points or sinusoidal patterns [9,10,21,22,23]. Specular reflections are inevitable and are often the source of errors in the estimated depths. To avoid the corrupted measurements, specular highlights can be: (1) removed in the image preprocessing step, (2) eliminated in multiview setups during the processing (the appearance of glints in images is view dependent; with the third or n-th view, every identified feature can be verified, and glints can be eliminated; the method is apt for scenes with single or few glints) [24], (3) filtered with the help of either polarized or chromatic filters (the filters are mounted in front of the camera lens; hence, there is no restriction on the number of present glints) [25] and (4) in industrial photogrammetry of rigid objects, attenuated through the use of special targets, e.g., doped with fluorescing dye, that respond to a wavelength other than the wavelength of the specular reflections [26].

Alternatively, the water being itself a source of infrared radiation can be observed with thermal cameras. Because the heat distribution is heterogeneous across the surface, it provides a good base for the correspondence search. A surface treated in this way can be measured with classical stereo- or multi-view photogrammetric approaches. If qualitative results are expected, it is sufficient to acquire single images and to proceed with data evaluation in the image space only [27].

#### 1.2.2. Water as a Specular Surface

Sometimes, it is advantageous to exploit the inherent optical characteristics of water, *i.e.*, the total reflection and refraction, for measurement purposes. Contaminating the liquid with physical material is cumbersome, because it becomes (1) unfeasible for large areas and field surveys, (2) difficult to keep a homogeneous point distribution and (3) it may influence the response of the water by interacting with it. In such situations, and depending on the working environment, whether in a lab or out in the field, it is possible to derive the surface shape by mere observation of a reflection of a source light or a pattern whose distortion corresponds to the surface slope and height

Reference [28] pioneered the characterization of water surface slopes with a reflection-based method. Their motive was to analyze slope distributions under different wind speeds through observing the Sun’s glitter on the sea surface from an aerial platform. Variations of the Cox and Munk method include: replacing the natural illumination with one or more artificial light sources, also known as reflective (stereo) slope gauge (RSSG) [3,29], and using entire clear or overcast sky to derive surface slope information for every point in the image, also known as Stilwell photography [30].

A combination of stereo- and reflection-based techniques (RSSG) was proven to be a sound way to characterize not only the surface slopes, but also the water heights. In a typical stereo setting, one is faced with a bias in the corresponding features seen by the left and the right cameras; the reason being that the cameras will record a change from the spots on the water whose normals’ are in the line of sight of the given cameras. Naturally, the steeper the observed wave, the less the systematic error. Using the Helmholtz reciprocity principle, *i.e.*, upgrading the method to employ light sources placed right next to the cameras, eliminates the correspondence ambiguity. The identified features in image spaces are then bound to be a unique feature in the object space [3,4].

In the field of computer vision, two principal classes of algorithms are shape from distortion and shape from specularity, which, e.g., inspect single or multiple highlights with a static or a moving observer and emitter [31,32,33], observe known or unknown intensity patterns reflected from mirror-like surfaces [34,35,36,37,38,39], directly measure the incident rays [40], exploit light polarization [41,42] and make assumptions on the surface’s bidirectional reflectance distribution [43,44,45]. Further interesting approaches that fall outside the scope of the adopted categories exploit other (than the visible) parts of the electromagnetic spectrum, such as infra-red [46], thermal [47] or UV. The principle resembles that of [48,49], *i.e.*, one searches for a wave spectrum, in which the information/signal received from the problematic surfaces, either doped or hit by the energy portion, is maximized, while minimizing the close-by, disturbing signals. For a good overview of the techniques, refer to [50,51].

#### 1.2.3. Water as a Refractive Medium

Refraction-based techniques are more complex due to the fact that the light path is dependent not only on the surface normal, but also the medium’s refraction index. The development of shape from refraction goes hand in hand with the shape from reflection methods and, therefore, has an equally long history. The work in [52], again, first experimented with light refraction to derive surface slopes from an intensity gradient emerging from beneath the water surface. Today, a successor of this technique, called imaging slope gauge, alternatively shape from refractive irradiance, is considered a highly reliable instrument for measuring wind-induced waves in laboratory environments. In contrast to the reflection-based techniques, refraction of a pattern through the water maintains a quasi-linear relationship between the slope and the image irradiance [14,53,54,55].

White light can also be replaced with active laser lighting. The laser offers a greater temporal resolution at the expense of a lower spatial resolution. The first examples of laser imaging gauges employed a single beam, thereby delivering information about a single slope [56,57]. Over time, they evolved to scanning systems that could capture spatial information over an area [58]. A laser slope gauge is used in the laboratory and field environment, however being most apt for the characterization of longer waves, as the literature reports.

In the field of computer vision, dynamic transparent surfaces have been analyzed with single- and multi-image approaches. The work in [59] first introduced a single view shape from the motion of a refractive body. By assuming that the water’s average slope is equal to zero, *i.e.*, it regularly oscillates around a flat plane, the author proposed a method for reconstructing the shape of an undulating water surface by inverse ray tracing. The work in [60] developed a method that recovers complete information of the 3D position and orientation of dynamic, transparent surfaces using stereo observations. The authors formulate an optimization procedure that minimizes a so-called refractive disparity (RD). In short, for every pixel in an image that refracts from an underwater pattern, the algorithm finds a 3D point on the water surface that minimizes the conceived RD measure. This measure expresses Snell’s law of refraction by enforcing that the normal vector of the computed 3D position must be equal when computed from the left and the right images of the stereo image. The two flag examples of computer vision approaches are limited to use in laboratory conditions due to (1) the need to locate a reference pattern under the water and (2) the demand of clear water for a reliable pattern to image correspondence retrieval. For more information, refer to [50,51].

## 2. Preliminaries

The traveling 3D sine wave is deemed (note that the shift term $H_{0}$ in Equation (2) is omitted; this is possible if the translation from GCS→LCS already compensates for that shift):

(1) $$y\left(t_{i}\right)=y_{i}$$

(2) $$z\left(t_{i}\right)=A\;\sin\left[\;2\pi \;\left(\frac{t_{i}}{T}-\frac{y_{i}}{\lambda }\right)+\phi \;\right],\;i=1\cdots N$$

where *ϕ* is the phase of the wave-front and *N* is the duration of the measurement in seconds. The traveling sine wave is a special surface, and as such, it is advantageously modeled in a *local coordinate system* (LCS), that is parallel to the wave propagation direction and shifted to the mean level of oscillations (*cf.* Figure 1 and Figure 6). In order to link the LCS with the *global coordinate system* (GCS) where the cameras are defined, a 3D spatial similarity transformation (the scale factor is unity; thus, the transformation reduces to a 3D rigid transformation) is formulated. The parameters of this transformation (*i.e.*, three components of the translation vector and three rotation angles) could likewise be included within the adjustment.

The methods invented devise the above-defined model in a least square approach, that is trying to minimize the residuals between the nominal observations and the observations predicted by that model. To solve the non-linear least squares problem, one must know the starting values of all parameters involved. Presented below is the strategy for retrieving the wave amplitude *A*, the wavelength *λ*, period *T*, phase-shift *ϕ* and the rigid transformation of the wave LCS to the camera GCS. Experience has shown that unless the amplitude is infinitesimally small and the wave infinitely long, even very rough estimates of the unknown wave parameters assure convergence of the system of equations.

Throughout the text, the notion of real and virtual points appears. The points are real if their projections in images come directly from the points (e.g., physical targets) or virtual (*i.e*., specular highlights) if the camera sees merely the reflection of a real point. Virtual points are always single-image observations, as their positions in space depend on the shape of the surface from which it is reflected and the view angle of the camera (*vide* the law of reflection).

### 2.1. Derivation of Approximate Wave Parameters

The wave amplitude can be: (i) estimated visually, on-site, during the measurement taking place, (ii) recovered from the wave probes, as these are a common place in any ship model basin, or (iii) derived from image-based measurement, provided there are observed real points found on the water surface (adopted by the authors). When the image-based approach is undertaken, the triangulation step must be followed to obtain the 3D coordinates of the real points (*cf.* Section 3.1.2). The amplitude can be recovered with the complex amplitude demodulation method (AMD) or merely by removing the trend from a point’s response and taking the halved maximum bound to be the starting *A* value. The collateral benefit of the AMD is that, were there is a varying amplitude signal, a slope instead of a horizontal line outcome would be observable. Accordingly, the *A* shall be replaced with a (linear) time-varying function, the parameters of which may be engaged in the total adjustment, as well.

Similarly, the value of the period *T* can be approximated either from on-site visual impressions, using the image data in post-processing (adopted by the authors), or taken directly from the wave probe data. The dominant period is then restored with the help of the spectral analyses, *i.e.*, the periodogram power spectral density estimate.

The wavelength might be inspected visually or with the help of the method presented in Section 3.1 (adopted by the authors). The wave probe devices, being single-point-based measurements, do not deliver enough data to indicate the length of the traveling wave.

The remaining wave parameter is the phase shift. Its computation requires a real point floating on the water surface, or otherwise, the wave probe data can become useful if its position is known in the reference coordinate system (CS) (adopted by the authors). If one moves the CS to that point, the term $\frac{y_{j}}{\lambda }$ in Equation (2) cancels out. If one further takes the starting frame $t_{1}=0$, also the term $\frac{t_{1}}{T}$ is gone, and the phase shift can be computed from $\phi =\arcsin\frac{z_{j}}{A}$, where *j* denotes the point at the origin of the translated CS. A slightly more elegant way to solve the equation for the initial phase shift is to use, again, the demodulation technique.

As for the wave transformation parameters ${\left(\omega ,\Phi ,\kappa ;T\right)}^{GCS\to LCS}$, one usually tries to define the global system that is quasi-parallel to the wave propagation direction or in best case that aligns with it. If so, the rigid transformation parameters can be set to zero values at the beginning of the adjustment and receive their corrections, which compensate for the inaccurate alignment. The image data and the scene context must be exploited to find the transformation relating the two coordinate system by identifying, e.g., the minimum of three common points or a 3D line and a point.

### 2.2. Optimization Technique

In the computational part, the Gauss–Markov least squares adjustment with conditions and constraints was adopted. The adjustment workflow proceeds in repetitive cycles of five steps, *i.e.*, (i) the generation of current approximate values of all unknown parameters, (ii) the calculation of the reduced observation vector, (iii) the calculation of the partial derivatives of the functional model with respect to all current parameters, (iv) the construction of the normal equations and (v) the solution of the system of equations.

The partial derivatives that make up the Jacobian matrix are always evaluated at the current values of the parameters. If the system of equations includes both condition and constraint equations, it does not fulfil the positive-definite requirement put by, e.g., the Cholesky decomposition. Indeed, on the diagonal of the equation matrix in Method 2, zero entries are present. The vector of solutions is then retrieved with the Gauss elimination algorithm.

## 3. Methods

### 3.1. Water as a Diffuse Surface

**Figure 2:**
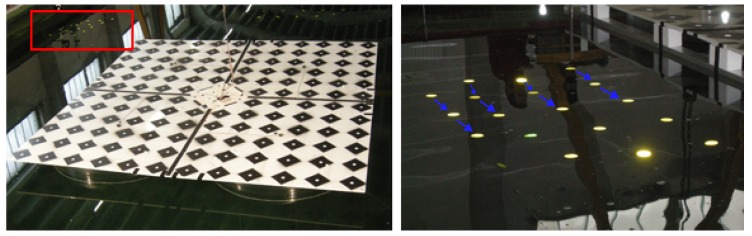
Left: physical, retro-reflective targets floating in the vicinity of the platform are marked with the red box. Right: a close-up of the targets; the arrows indicate established cluster pairs.

The following method sees the water as a diffuse surface. It is converted to such, owing to artificial targeting. A set of retro-reflective targets floated on the water surface were tracked during the measurements (*cf.* Figure 2). The targets were in-house produced using a diamond grade reflective sheeting (3M${}^{TM}$, Minneapolis, Minnesota, USA). The sheet, thanks to the cube corner reflectors’ composition, allowed for an efficient light return for very wide entrance angles, thereby assuring good visibility in the oblique looking camera views. The targets were interconnected with a string, so as to avoid their collision and dispersion; their spacing equaled *ca*. 30 cm.

Reconstruction of a singular point provides for all but one wave parameter: the wavelength *λ*. Combining the responses of the minimum of two such points allows for the possibility of recovering also the remaining *λ*. It is presumed that the 3D data have already been transformed to the *LCS*, and to shorten the discussion, this is excluded from the mathematical model. A complete description on how to include this information in the model is given in Section 3.2.

Since the parameters are found in a least squares approach, the discussion commences with the initial parameter retrieval. In the next step, the adjustment mathematical model is outlined. Evaluation of the results continues in Section 5.

#### 3.1.1. Mathematical Model

**Figure 3:**
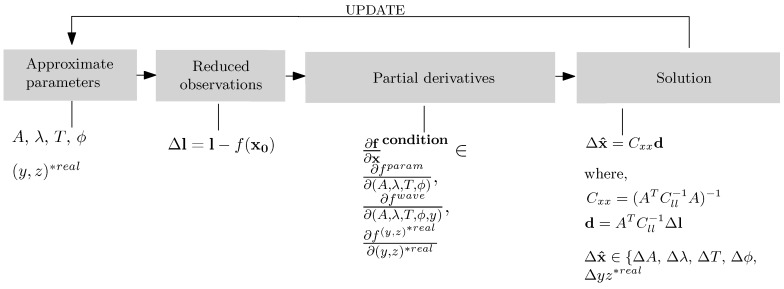
The least squares adjustment workflow of Method 1. The cycle repeats until the stop criterion is reached. ∂x denotes the entire vector of parameters; *A* is the Jacobian in observation equations; and $C_{ll}$ is the covariance matrix. See $f^{wave}$, $f^{param}$ in Equation (2) and Equation (6).

The functional model describes analytically the prior knowledge on the wave shape expressed in Equation (2). The modeling part, unlike in Method 2 in Section 3.2, is formulated purely in 3D space. The point triangulation is treated as a separate and unrelated phase, even though the image measurements indirectly contribute to the eventual outcome, and a joint treatment might be suggested. As a matter of fact, the 3D space is reduced to a 2D space, building on the fact that the transformation from *GCS* to *LCS* has taken place in advance, and the *x*-coordinate can take arbitrary values. All parameters are present in the adjustment as observations and unknowns; see the adjustment workflow in Figure 3. The stochastic model is formalized within the weight matrix and conveys the observation uncertainty. The matrix holds non-zero elements on its diagonal, and they may take the following form: $w_{i}=\frac{\sigma_{0}^{2}}{\sigma_{i}^{2}}$, where $\sigma_{i}$ signifies the *a priori* standard deviation of an observation and $\sigma_{0}$ is the *a priori* standard deviation of a unit weight. Within the experiments, the $\sigma_{0}$ was set to unity, whereas $\sigma_{A}=2$ mm, $\sigma_{T}=0.05$ s, $\sigma_{\lambda }=100$ mm, $\sigma_{\phi }=0.25$ rad. These values are rough and rather pessimistic estimates of the uncertainty of the approximate parameters.

The condition equations are formed by all parameters that are regarded as observed, *i.e*., $y_{i}$, $z_{i}$, *A*, *T*, *λ*, *ϕ*, and follow Equation (6). Every observed point *i* provides three condition equations: ${\hat{y}}_{i}=y_{i}$, ${\hat{z}}_{i}=z_{i}$ and Equation (2).

#### 3.1.2. Image-Based Approximate Wave Retrieval

The starting point is to measure the targets in images, for instance with centroiding methods, ellipse fitting or cross-correlation [61,62,63]. The authors adopted the intensity centroiding method to detect points in the initial frame and the sub-pixel cross-correlation to track them in time [64]. The 2D image measurements are then transferred to 3D space in a regular bundle adjustment and exploited to recover the initial wave parameters. The developed pipeline is fully automatic and summarized in the following order:

1. clustering of 3D points;
2. coupling of neighboring clusters;
3. calculation of mean *A*, *T* and *ϕ* from the clusters;
4. calculation of mean *λ* from the couples of clusters.

The reconstructed targets in time are considered an unorganized point cloud; thus, their clustering is carried out up front (*cf.* Figure 2). The retrieved clusters are equivalent to the responses of a single target floating on the water surface. The clustering *per se* is not required, as this piece of information is already carried within the point naming convention. Nonetheless, because this may not always be the case, the clustering is a default operation. It creates boundaries in 3D space based on some measure of proximity; in our case, the Euclidean measure was chosen. The algorithm was invented by [65] and implemented in [66].

Now, the coupling establishes a neighborhood relationship between closest clusters (*cf.* Figure 2). Given a *kd*-tree representation of all clusters’ centroids, the algorithm searches for the neighbors within a desired radius and ascertains that the selected pair has an offset along the wave propagation direction. This later permits for the computation of the wavelength *λ*. The selected radius should not be too small, but cannot be greater than the wavelength, to be able to resolve its length.

**Figure 4:**
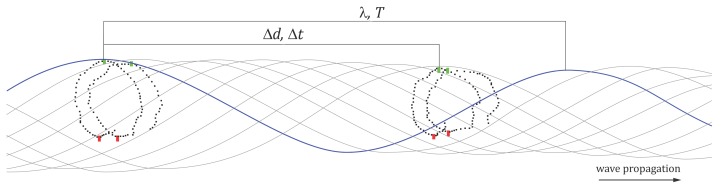
Image-based approximate wave retrieval. For this cluster pair, the wave crest takes eight frames (Δt) to travel between the neighbors. If the distance between the clusters is Δy=10 cm and the period T=12, then the wavelength λ=15 cm.

The mean *A*, *T* and *ϕ* are found within each single cluster, as pointed out in Section 2.1. To find the mean *λ*, the requirement is to know: (i) the period *T*, (ii) the direction of the wave propagation, and (iii) the relation between the *GCS* and the *LCS*, where the wave is defined. For every couple of clusters, one counts how much time Δt a wave crest takes to travel between the clusters (*cf.* Figure 4). The distance Δd (in *LCS*) between them is known and so is the period *T*, hence the wavelength estimate results from the trivial proportion:

(3) $$\frac{\Delta d}{\Delta t}=\frac{\lambda }{T}$$

### 3.2. Water as a Specular Surface

**Figure 5:**
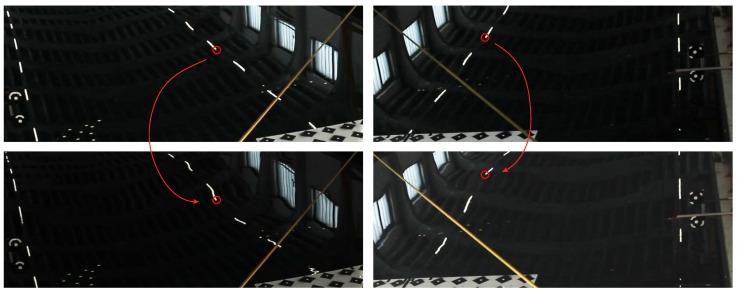
Specular reflections of the control information seen by two cameras. **Top**: calm water condition; the water can be considered a plane, and linear features map to linear reflections. **Bottom**: water in the excited condition; linear features map to deformed reflections.

The second developed method exploits the fact that water under some conditions can be perceived as specularly reflecting, *i.e.*, manifesting no scattering on its surface. Two parallel arrays of linear lamps hang above the measurement space (*cf.* Figure 8). Their reflections on top of the water surface could be observed as static, in the steady wave condition, and dynamic, when the water was excited. In the latter case, the reflections project to distorted shapes (*cf.* Figure 5). Such deformations implicitly carry information about the instantaneous shape of the water surface and are investigated in the successive paragraphs.

Because the specularities (also known as highlights) travel with the observer and depend on the surface geometry, no corresponding features in different cameras are present [33,51,67]. As a result, no stereo or multi-view measurements are made possible. Unless one is able to directly identify 3D rays that would intersect at the interface of a surface [40], the alternative solution to the depth-normal ambiguity is to add control information and/or impose appropriate constraints in 3D object space.

In the developed approach, the images of specular highlights and a number of parameter constraints are combined together to recover the water’s instantaneous state. This method solves a least squares problem, simultaneously determining all parameters of interest. The discussion opens with the condition equations and imposed constraints, which constitute the functional model of the LS problem. Next, the adjustment procedure is explicitly given, including: (i) the derivation of approximate values for all unknowns, (ii) the stochastic model, (iii) the system equation forming, as well as (iv) the collection of control information. Lastly, the experimental section presents the results followed by a compact conclusive paragraph.

#### 3.2.1. Mathematical Model

The functional model comprises the mathematical description of two observed phenomena, that is the perspective imaging associated with the camera system, as well as the shape of the induced waves, which in turn associates with a wave maker.

The camera to object points relation was modeled with the collinearity equations. The shape of the induced waves was modeled with Equation (2). The defined wave model is accompanied by three constraint equations. They impose that: (i) virtual points lie on the wave surface ($f_{surf}$) (their distance from the surface = 0), (ii) for all virtual points, the incident and reflected rays make equal angles with respect to the tangent/normal at that point ($f_{refl}$) (compliance with the law of reflection), and (iii) the vector from the camera to a virtual point, the normal at the point and the vector from that point towards its 3D real position are coplanar ($f_{copl}$) (compliance with the law of reflection). The real points are always considered as ground control information; therefore, the developed method belongs to the class of calibrated environment methods. See the adjustment workflow in Figure 7.

Apart from what has been so far discussed in Section 3.1.1, the stochastic model avoids having the solution driven by the observations that are most abundant. It limits the influence of a particular group of observations with the help of a second weighting matrix $N_{max}$. Here, every group of observations was assigned a value $n_{max}$ that limits its participation in the adjustment to below $n_{max}$ observations. The diminishing effect is realized by the expression in Equation (4) and found on the diagonal of the matrix. The $n_{obs}$ is the cardinality of the observations within a group. The ultimate weight matrix $W^{\prime }$ is a multiplication, $W^{\prime }=W\cdot N_{max}$. The bespoke weighting strategy is implemented within MicMac, an open source bundle adjustment software [68,69].

(4) $$n_{max}^{obs,i}=\frac{\frac{n_{obs}\;n_{max}}{n_{obs}+n_{max}}}{N_{obs}},\;i=1\cdots N$$

Within the experiments, the $\sigma_{0}$ value was always set to unity, whereas $\sigma_{A}=1$ mm, $\sigma_{\lambda }=10$ mm, $\sigma_{T}=0.05$ s, $\sigma_{\phi }=0.25$ rad, $\sigma_{xy}=0.5$ pix, $\sigma_{XYZ}^{real}=5$ mm, $\sigma_{XYZ}^{virtual}=10$ mm, $\sigma_{T}^{GCS\to LCS}=25$ mm and $\sigma_{\omega ,\phi ,\kappa }^{GCS\to LCS}=0.01$ rad. In analogy to Method 1, these values are rough estimates of the estimated approximate parameters’ values. If the parameter setting shall be unclear, a means of assessing the correctness of the *a priori* values must be employed, e.g., the variance component analysis.

**Figure 6:**
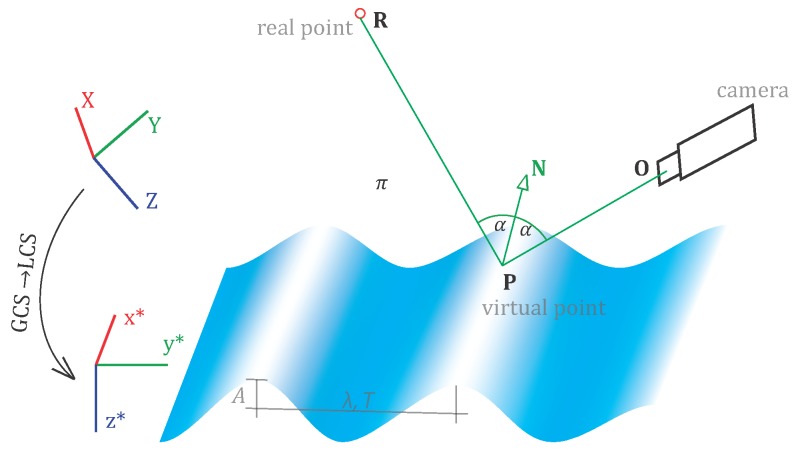
Graphical representation of the functional model. The camera captures the scene following the laws of perspective projection. The wave model, displayed at a time instance, is defined through parameters *A*, *λ*, *T* and *ϕ* in LCS. The camera (*O*), the virtual point P and real point R lie on a common plane *π*, whereas the incident and reflecting rays are symmetric *w.r.t.* the normal N.

**Figure 7:**
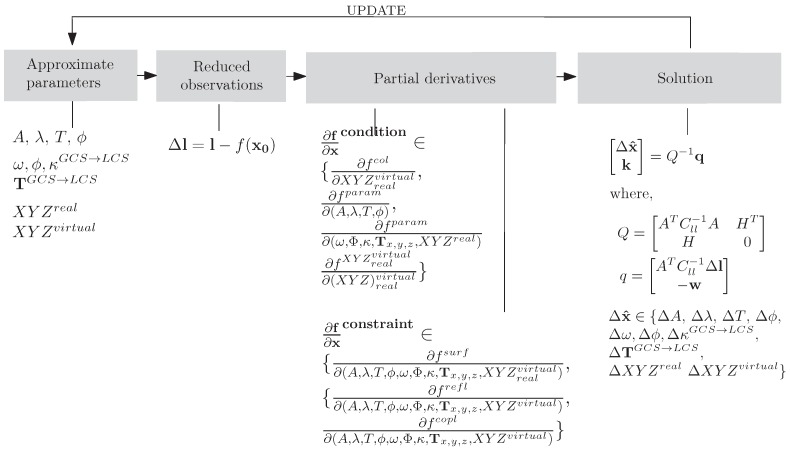
The least squares adjustment workflow of Method 2. The cycle repeats until the stop criterion is reached. ∂x denotes the entire vector of parameters; *A* is the Jacobian in observations equations; *H* is the Jacobian in constraint equations; *k* is the Lagrange multiplier; and $C_{ll}$ is the covariance matrix. See $f^{param}$, $f^{surf}$, $f^{refl}$, $f^{copl}$ in Equations (6)–(9), respectively.

Condition equations are functions of observations and parameters. Collinearity equations are self-evidently observations as a function of parameters: the 3D coordinates of the real or virtual point (IOR and EOR in the developed implementation were treated as constants). Equation (5) renders the collinearity expanded into Taylor series around the *N* initial estimates $XYZ_{i}^{0}$:

(5) $$f_{\left(XYZ\right)}^{col}=f^{col}\left(XYZ_{i}^{0}\right)+\frac{\partial f^{col}}{\partial {\left(XYZ\right)}_{i}^{0}}\;\;d{\left(XYZ\right)}_{i},\;i=1\cdots N$$

Optionally, one may define originally free parameters as observed unknowns. This trick helps to include any available knowledge of the unknowns into the pipeline, as well as to avoid surplus parameter updates, *i.e.*, steer the rate of parameter change along the iterations. The parameters to control the rate are the entries of the weight matrix *W*. Our implementation allows all parameters to be regarded as observed; therefore, any parameter in Figure 7 can be replaced with the param in Equation (6). For instance, if an *X*-coordinate is observed, the condition equation $\hat{X}=X^{obs}+v_{x}$ and the correction equation $X=X^{obs}+1\cdot dX$ are written down, where $v_{x}$ and dX are the observation and the parameter corrections.

(6) $$f^{param}=f_{0,i}^{param}+\frac{\partial f^{param}}{\partial {\left(param\right)}_{i}^{0}}\;\;d{\left(param\right)}_{i},\;i=1\cdots N_{param}$$

Constraint equations do not involve observations, but pure parameters. The conceived wave model renders three constraint equations:

(i) $f^{surf}$,
  (7) $$f^{surf}=-z^{*}\left(t_{i}\right)+A\;\sin\left[\;2\pi \;\left(\frac{t_{i}}{T}-\frac{y_{i}^{*}}{\lambda }\right)+\phi \;\right]=0,$$
(ii) $f^{refl}$,
  (8) $$f^{refl}={\mathrm{ff}}^{OP}-{\mathrm{ff}}^{PR}=\arccos\left(\frac{\vec{OP}\;\vec{N}}{|\vec{OP}\left|\;\right|\vec{N}|}\right)-\arccos\left(\frac{\vec{PR}\;\vec{N}}{|\vec{PR}\left|\;\right|\vec{N}|}\right)=0$$
(iii) $f^{copl}$,
  (9) $$f^{copl}=\vec{OP}\;\left(\vec{PR}\times \vec{N}\right)=\left|\begin{array}{ccc} OP_{x*} & OP_{y*} & OP_{z*}\\ RP_{x*} & RP_{y*} & RP_{z*}\\ N_{x*} & N_{x*} & N_{x*} \end{array}\right|=0$$

where $\vec{OP}=\left[x_{O}^{*}-x_{P}^{*}\;y_{O}^{*}-y_{P}^{*}\;z_{O}^{*}-z_{P}^{*}\right]$, $\vec{PR}=\left[x_{R}^{*}-x_{P}^{*}\;y_{R}^{*}-y_{P}^{*}\;z_{R}^{*}-z_{P}^{*}\right]$, $y^{*}\;=\;y^{*}\left(X,Y,Z,\omega ,\Phi ,\kappa ;T\right)$ and $z^{*}=z^{*}\left(X,Y,Z,\omega ,\Phi ,\kappa ;T\right)$. The constraints are defined locally; thus, coordinate quantities are annexed with the symbol ${}^{*}$. The values determined in LCS are not considered in the adjustment, but are obtained after a 3D rigid transformation with the parameters ${\left(\omega ,\Phi ,\kappa ;T\right)}^{GCS\to LCS}$.

The linearized forms of the above equations, expanded into Taylor series, are presented in Appendix A. Note that the local coordinate quantities $x^{*},y^{*},z^{*}$ are functions of their positions X,Y,Z in the GCS, as well as the parameters of the 3D rigid transformation. As a result, the derivatives are calculated for a composition of functions and must obey the chain rule.

#### 3.2.2. Derivation of Control Information

**Figure 8:**
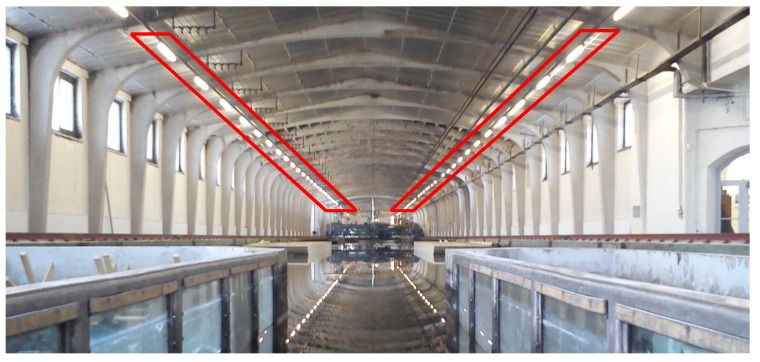
Two arrays of lamps are marked with red bounding boxes. The end points of the single lamps served as the control information.

**Figure 9:**
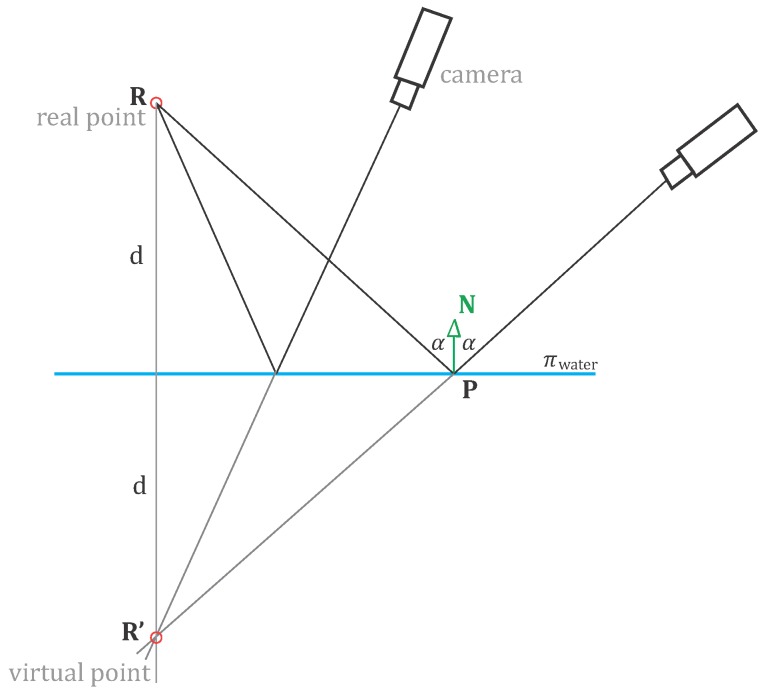
The imaging configuration taking place when deriving the control information. R represents the end-point of a linear lamp; $R^{\prime }$ is its virtual location, at *d* distance behind the water plane *π*. The camera to real-point rays follow the law of reflection, *i.e*., the incident and reflecting rays form equal angles with the plane normal N.

The control information was not acquired physically prior to nor during the measurements. Not even posterior efforts were undertaken to collect the ground truth. The position of the linear lamps (*cf.* Figure 8), which served as the ground truth information, was recovered solely using the image data, under the condition that the reflecting water is globally a plane. As each measurement started with the calm water condition, the planarity condition was valid at numerous times. The imaging situation is depicted in Figure 9.

The calculation of the XYZ coordinates of the control information in their real locations divides into: (i) the water plane derivation (from real points), (ii) the identification of homologous points across views and triangulation (virtual points), and lastly (iii) the flipping of the virtual points to their real positions.

The plane *π* of the water was recovered thanks to well-distributed dust particles present on its surface. Their appearance was sufficiently discriminative for their identification across views. Alternatively, one could place artificial targets on top of the water to avoid potential identification problems. Given a few (≥3) pairs or triples of 2D image points corresponding to real features, their 3D position is found by intersection. The searched plane defined analytically as Ax+By+Cz+D=0 is then established by singular value decomposition (SVD).

The end points of the linear lamp reflections were identified and measured in images manually only in the initial frame. The subsequent tracking in time was realized by the flagship cross-correlation technique. Having found and measured the reflections, their 3D locations are triangulated ($R^{\prime }$ in Figure 9), ignoring the fact that the observed features are not real. The 3D points emerge on the wrong side of the water plane; thus, they must be flipped to their real positions. The flipping is done with respect to an arbitrary plane, being the water plane and determined by the coefficients A,B,C and *D*. The transformation performing that operation works by:

(i) roto-translating the global coordinate to a local coordinate system that aligns with the flipping plane,
  (10) $$\begin{array}{c} T\cdot R_{1}=\left[\begin{array}{cccc} 1 & 0 & 0 & 0\\ 0 & 1 & 0 & 0\\ 0 & 0 & 1 & 0\\ -X_{i} & -Y_{i} & -Z_{i} & 1 \end{array}\right]\cdot \left[\begin{array}{cccc} \frac{\lambda }{|N|} & 0 & \frac{n_{x}}{|N|} & 0\\ \frac{-n_{x}n_{y}}{\lambda |N|} & \frac{n_{z}}{\lambda } & \frac{n_{y}}{|N|} & 0\\ \frac{-n_{x}n_{z}}{\lambda |N|} & -\frac{n_{y}}{\lambda } & \frac{n_{z}}{|N|} & 0\\ 0 & 0 & 0 & 1 \end{array}\right] \end{array}$$
  where N = [$n_{x}$$n_{y}$$n_{z}$], *λ* = $\sqrt{n_{y}^{2}+n_{z}^{2}}$, and [$X_{i}$$Y_{i}$$Z_{i}$] are 3D coordinates of any points lying within the flipping plane.
(ii) performing the actual flipping over the local XY-plane:
  (11) $$\begin{array}{c} R_{2}=\left[\begin{array}{cccc} 1 & 0 & 0 & 0\\ 0 & 1 & 0 & 0\\ 0 & 0 & -1 & 0\\ 0 & 0 & 0 & 1 \end{array}\right] \end{array}$$
(iii) and bringing the point back to the global coordinate system with $R_{1}^{-1}$ and $T^{-1}$.

The entire procedure committed in a single formula renders:

(12) $$M=T\cdot R_{1}\cdot R_{2}\cdot R_{1}^{-1}\cdot T^{-1}$$

#### 3.2.3. Derivation of the Approximate Highlight Position

The highlights’ coordinates (P in Figure 6) result from the intersection of the approximate wave model with the vectors anchored in the image measurements, passing through the camera perspective center and extending into the object space. The highlights are single-image observations, so there exist no correspondences across images, as in the case of real points. The intersection points are found first by intersecting with the mean water plane and then by iteratively improving the results with Newton’s method. The points are first found in the LCS and subsequently transferred to the GCS given the approximate parameters of the rigid transformation. The algorithm is presented below.

Given the 3D vector defined by points $\left(x_{1}^{*},y_{1}^{*},z_{1}^{*}\right)$ and $\left(x_{2}^{*},y_{2}^{*},z_{2}^{*}\right)$ at the camera center and observed in image space, respectively, the 3D line parametric equation takes the form:

(13) $$\left[\begin{array}{c} x^{*}\\ y^{*}\\ z^{*} \end{array}\right]=\left[\begin{array}{c} x_{1}^{*}\\ y_{1}^{*}\\ z_{1}^{*} \end{array}\right]+t\cdot \left[\begin{array}{c} x_{2}^{*}-x_{1}^{*}\\ y_{2}^{*}-y_{1}^{*}\\ z_{2}^{*}-z_{1}^{*} \end{array}\right]$$

The sought $y^{*}-$coordinate of the intersection is then equal to $y^{*}=y_{1}^{*}+t\cdot \left(y_{2}^{*}-y_{1}^{*}\right)$ where $t=\left(z^{*}-z_{1}^{*}\right)/\left(z_{2}^{*}-z_{1}^{*}\right)$. The unique solution can be obtained when the *z* in the preceding equation is replaced with the mean water level, e.g., $H_{0}=0$ in the LCS. A better approximation can be accomplished if the intersection is performed with a more realistic model than the plane: the observed sine wave. Combining Equation (2) with the last row of Equation (13), such that the $z^{*}$ terms are equal, brings about the following relationship:

(14) $$g\left(y^{*}\right)=y^{*}-y_{1}^{*}-\frac{\left(y_{2}^{*}-y_{1}^{*}\right)}{\left(z_{2}^{*}-z_{1}^{*}\right)}\cdot \left(H_{0}+A\;\sin\left[\;2\pi \;\left(\frac{t}{T}-\frac{y^{*}}{\lambda }\right)+\phi \;\right]-z_{1}^{*}\right)=0$$

The function *g* has one parameter $y^{*}$, and as such, together with its derivative $g^{\prime }$, both evaluated at the current parameter value $y_{0}^{*}$, they enter Newton’s method, which finds the ultimate root. The Newton step is the ratio of $g(y_{0}^{*})$ and $g^{\prime }\left(y_{0}^{*}\right)$ (*cf.* Equation (15)). The loop continues until the difference between old and new parameter estimates no longer falls below a defined threshold.

(15) $$y^{*}=y_{0}^{*}-\frac{g(y_{0}^{*})}{g^{\prime }\left(y_{0}^{*}\right)}$$

Once the $y^{*}$ value is known, the $z^{*}$-coordinate is computed from Equation (2), and lastly, the $x^{*}$ can be retrieved from the 3D line equation as $x^{*}=x_{1}^{*}+\frac{z^{*}-z_{1}^{*}}{z_{2}^{*}-z_{1}^{*}}\cdot \left(x_{2}^{*}-x_{1}^{*}\right)$. The final step brings the locally-determined coordinates to the global ones with ${\left(\omega ,\Phi ,\kappa ;T\right)}^{GCS\to LCS}$.

## 4. Imaging System

The imaging setup is comprised of three dSLR cameras (Canon 60D, 20-mm focal length) and three continuous illumination sources (1250 W). The spatial resolution of the videos matched the full HD (1920 × 1080 pix), acquiring a maximum of 30 fps in progressive mode. The video files were lossy compressed with the H.264 codec and saved in a .mov container.

**Figure 10:**
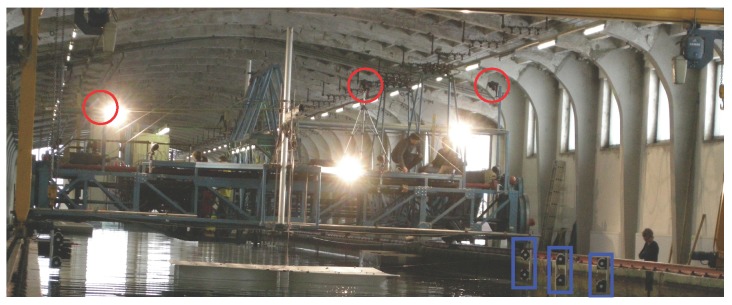
Red circles point to three cameras placed on a moving platform, across the model basin. The blue rectangles point to three scale bars. The remaining scale bars are symmetrically arranged on the opposite side of the basin.

**Figure 11:**
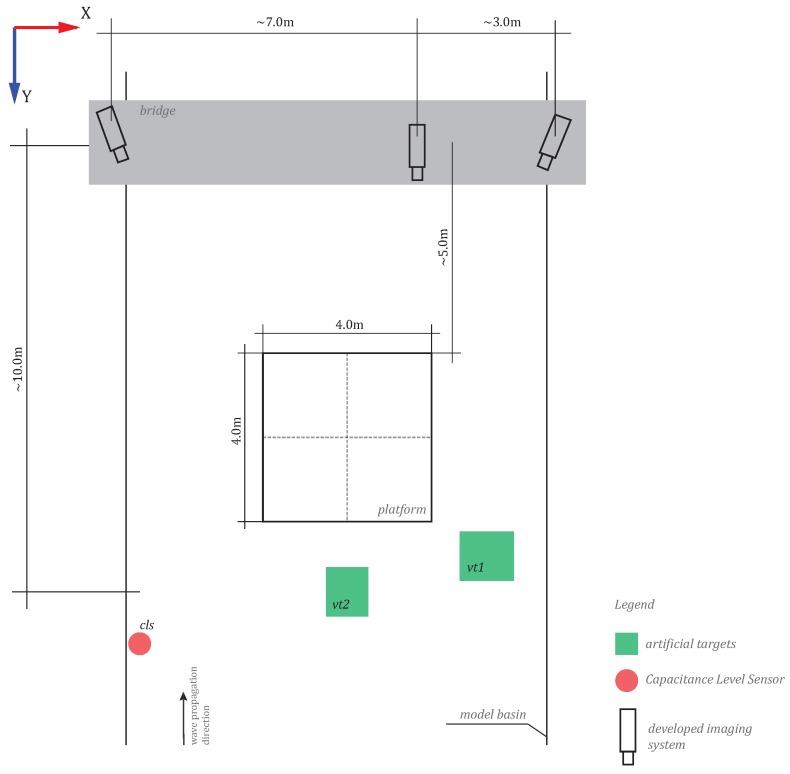
The top view of the measurement workplace. Cameras on top of the drawing are placed on a mobile bridge. The platform occupies the scene’s central part and is located *ca*. 5 m away from the bridge. The green rectilinear shapes correspond to the zones of the artificial targets (used in Method 1, and in the evaluation of Methods 1 and 2; see Section 3.1 and Section 5), while the red circle is the capacitive level sensor (used in the evaluation of Methods 1 and 2; see Section 5).

The mean object to camera distance amounted to 10 m, resulting in the average image scale of 1:500. The cameras were rigidly mounted on a mobile bridge (*cf.* Figure 10 and Figure 11) and connected with each other, as well as with a PC, via USB cables to allow for: (i) remote triggering, (ii) coarse synchronization. Fine-alignment of the video frames was possible with the help of a laser dot observed in all cameras. The laser worked in a flicker mode, at a frequency lower than that of the video acquisition, and casually moved over the floating platform’s surface, both at the start and the finish of each acquisition. No automatization was incorporated at this stage; instead, the alignment was conducted manually.

Despite the USB connections, the videos were stored on the memory cards. No spatial reference field was embedded in the vicinity of the system; instead, the calibration and orientation was carried out with the moved reference bar method [70].

## 5. Experiments

### 5.1. Evaluation strategy

Results achieved with Method 1 (*m1*) and Method 2 (*m2*) are confronted with the responses of a capacitive level sensor and validating physical targets (*cf.* Figure 11). The capacitive level sensor was mounted on a rod-like probe and sensed the variations in electrical capacity within the sensor. Given the dielectric constant of the liquid, this information can be directly transformed to the changes in the water level, in which the probe is normally immersed. Because the sensor samples the changes in a singular spot, it provides merely information on the amplitude and frequency of the water level oscillations; likewise validating the targets. The instantaneous wavelength, thereby, remained unknown, as no direct means to judge the accuracy of the calculated wavelength existed. Indirectly, the correctness of all wave parameters, including the wavelength, can be estimated by confronting the response of a number of points distributed along the wave propagation (vt1,vt2,cls) with their responses predicted from the model.

**Figure 12:**
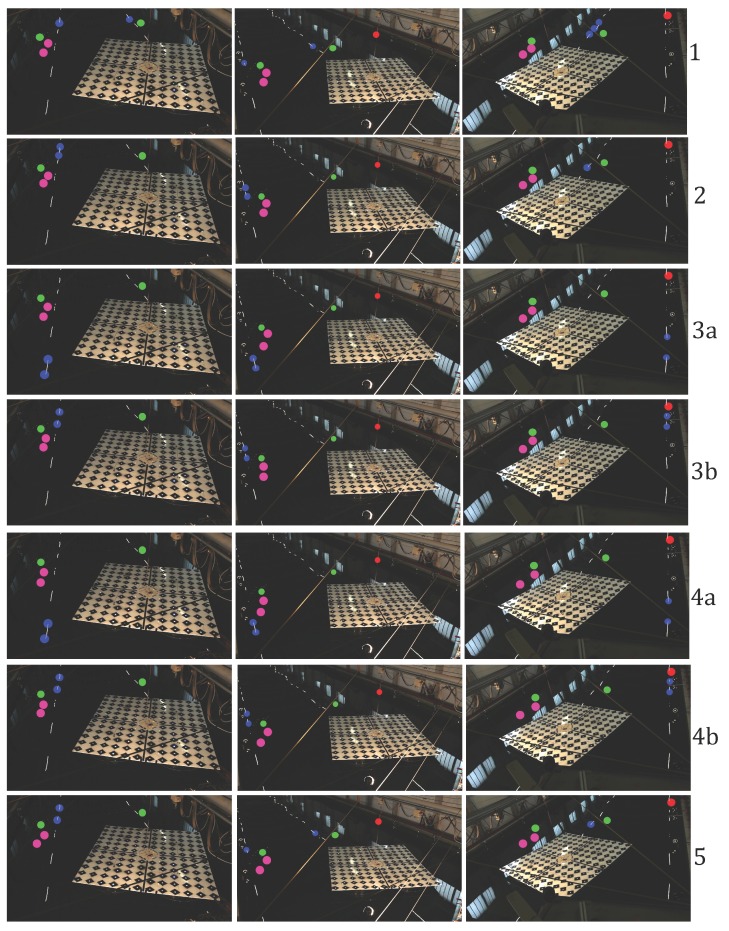
Distribution of observed highlights (blue) used in *m2*, artificial targets used in *m1* (magenta), validating targets (green) and the capacitive level sensor (red) in three camera views, in all measurement series.

**Figure 13:**
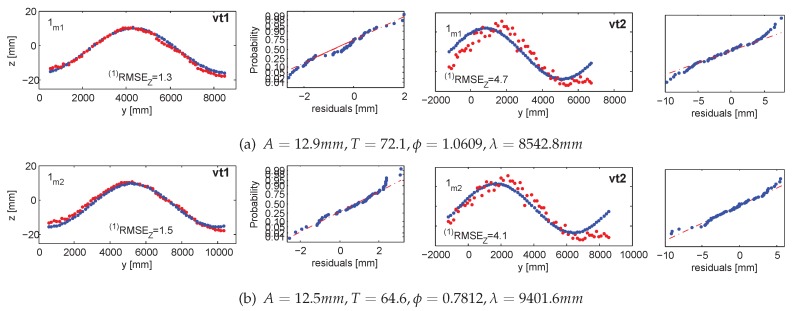
Accuracy 1 validation results in the first measurement series for m1 in (**a**) and m2 in (**b**), compared against the response of vt1 and vt2. Ground truth in red; fitted wave model in blue. The normal probability plots are in even columns.

**Figure 14:**
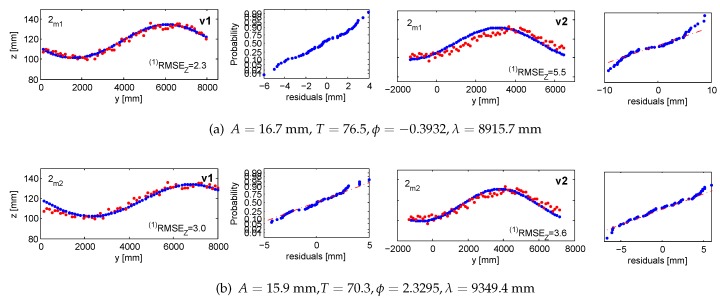
Accuracy 1 validation results in the second measurement series for m1 in (**a**) and m2 in (**b**), compared against the response of vt1 and vt2. Ground truth in red; fitted wave model in blue. The normal probability plots are in even columns.

**Figure 15:**
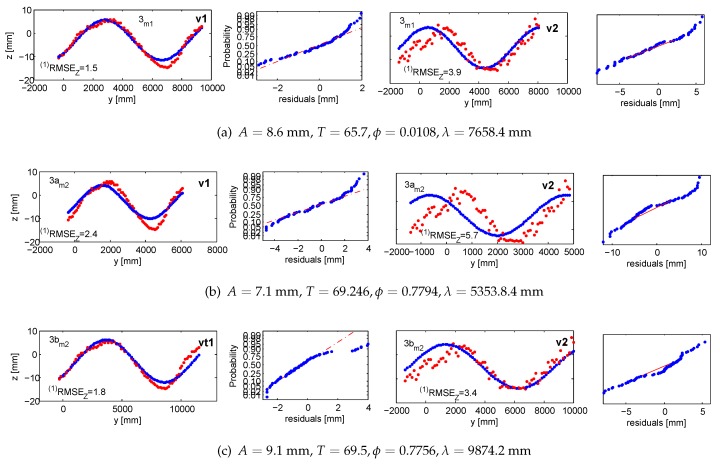
Accuracy 1 validation results in the third measurement series for m1 in (**a**) and m2 in (**b**) and (**c**), compared against the response of vt1 and vt2. Ground truth in red; fitted wave model in blue. The normal probability plots are in even columns.

**Figure 16:**
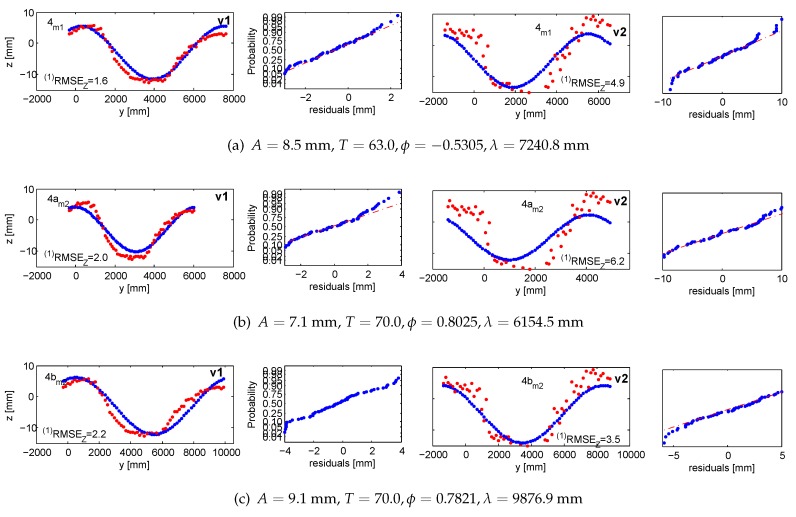
Accuracy 1 validation results in fourth measurement series for m1 in (**a**) and m2 in (**b**) and (**c**), compared against the response of vt1 and vt2. Ground truth in red; fitted wave model in blue. The normal probability plots are in even columns.

**Figure 17:**
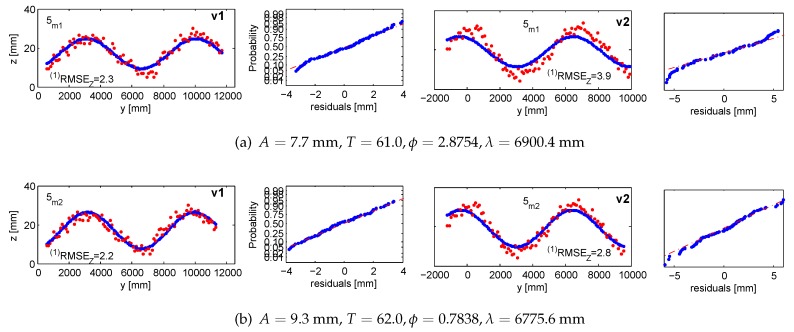
Accuracy 1 validation results in the fifth measurement series for m1 in (**a**) and m2 in (**b**), compared against the response of vt1 and vt2. Ground truth in red; fitted wave model in blue. The normal probability plots are in even columns.

**Figure 18:**
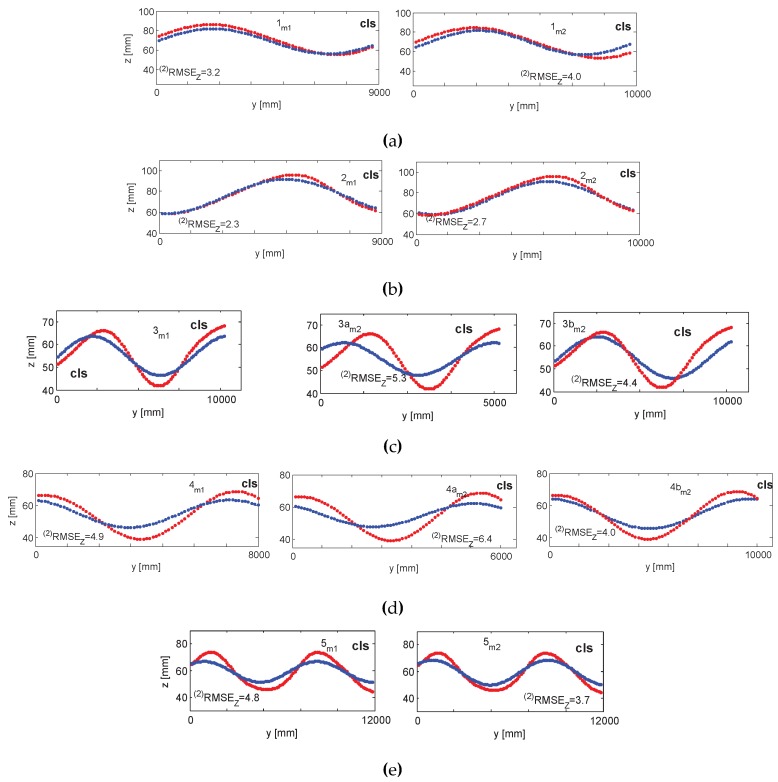
Accuracy 2 validation results for m1 and m2 in the 1–5 measurement series depicted in **a**–**e**, respectively. In red, the cls response; in blue the m1, m2 responses. All comparisons are carried out at the position of point cls.

In the adjustment, *m1* adopted three clusters, whereas *m2* tracked up to seven highlights, corresponding to four to six ground control points (*i.e.*, the lamps’ endpoints). The distribution of measured and validating points is displayed in Figure 12. Numerical results of the five measurement series are summarized in Table 1, with the graphical representations provided in Figure 13, Figure 14, Figure 15, Figure 16, Figure 17 and Figure 18. The third and fourth measurement series was evaluated twice with varied image observations (specular highlights; *cf.* Figure 12). The adopted evaluation strategy is as follows.

#### 5.1.1. Accuracy 1: Validating Targets (vt1, vt2)

With validating targets, we refer to points that were not treated in the adjustments aiming at finding the wave parameters. They were measured in images and independently intersected in 3D space. The *Z*-response of all validating targets is confronted with the value predicted from the devised wave model. The validation takes place in the *LCS*. Figure 13 and Figure 14 illustrate the results projected onto the traveling sine wave. The red corresponds to the response from the target; the blue is the model outcome. The normal probability plots test and confirm that the residuals follow the normal distribution.

#### 5.1.2. Accuracy 2: The Capacitive Level Sensor (cls)

To compare the data collected by the capacitive level sensor and the image-based measurement system, temporal synchronization and data resampling had to take place. The start of the capacitive level sensor (cls) data collection was conveyed to the cameras audiovisually, by switching the light on and by emitting vocal sound. This allowed for rough temporal synchronization. To fine align the two signals, cross-correlation was carried out. The frequency of cls data collection was double the frequency of the camera recording; therefore, to equalize the acquisition rates, every other sample of the cls device was discarded. Figure 18 illustrates the results of the comparisons.

**Table 1:** Mean precision ($\sigma_{x,y,z}$) and accuracy (${}^{\left(1\right)}RMSE_{z}$, ${}^{\left(2\right)}RMSE_{z}$) of five measurement series evaluated with Method 1 and Method 2.

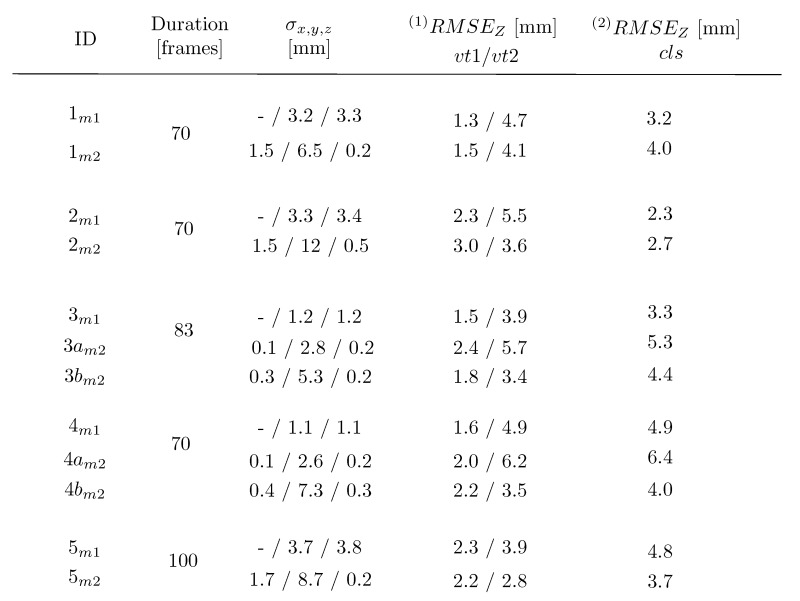

### 5.2. Discussion

The results achieved were confronted with the responses of a capacitive level sensor and two validating targets, all of which provided single-point responses, and were placed in various positions across the water basin. In an overall assessment, the specular method (Method 2) proved superior with respect to the diffuse method (Method 1).

#### 5.2.1. Accuracy

Method 1 performs well locally, when validated on points in the vicinity of the cluster pair (vt1), but as soon as it is confronted with distant points (vt2), modeling errors significantly grow; compare, e.g., Figure 13a at vt1 and vt2. Method 2 employs the entire water field in the computation and therefore has a global scope with the absence of extrapolation effects, and the modeling errors are more consistent, yet have a slightly higher magnitude. It shall be noted that the vt1 and cls placed on either end of the basin were under the influence of the principal sine wave, as well as the waves reflected from the basin side walls. The platform floating in the middle also disturbed the principal wave shape. The third and fourth measurement series evaluated with Method 2 on the highlights observed at the top of the basin (series $3a_{m2}$ and $4a_{m2}$) and around the platform (series $3b_{m2}$ and $4b_{m2}$) proved that the wave, having faced an obstacle, decreases its amplitude and wavelength. Compared the significant deviations of the blue/red curves in Figure 15b and Figure 16b at vt2, as well as Figure 18c,d at the cls, as opposed to Figure 15c and Figure 16c and Figure 18c,d respectively. This is of high importance in interpreting the behavior of the platform. To do that, one must know the form of the water body just before it hits the platform and not some distance before that interaction, since it no longer corresponds to the real force put on that object.

Evaluation results on cls suggest that the wave form changed spatio-temporally. It was systematically attenuated with increasing distance from the generating source. The cls was mounted closer to the wave maker than vt1, vt2, other artificial targets or the highlights and, consequently, measured higher wave amplitudes; compare the subfigures of, e.g., Figure 18c or d. Wave superposition effects (the principal and reflected waves) could contribute to higher amplitudes, as well.

#### 5.2.2. Precision

Precision measures should not be interpreted as a single quality measure. As the evaluation proved, they are too optimistic when confronted with the accuracy measures. Moreover, the covariance matrices in Method 1 return a standard deviation homogeneous in all coordinates, while Method 2 manifests large uncertainty in the *y*-coordinate. This is due to the simplified and rigorous modeling of Methods 1 and 2, respectively. Method 2 simultaneously treats the reconstruction and the modeling task, whereas Method 1 performs merely the modeling, with no special treatment of the preceding steps, other than the known *a priori* standard deviation expressed in the weight matrix.

The inferior precision on the *y*-coordinate in Method 2 is a side effect of suboptimal network design, with a good base across the model basin (*x*-coordinate) and practically no shift along the *y*-axis (see the definition of the coordinate system in Figure 11). In spite of no parallax on the *z*-coordinate, the precision figures along that axis are satisfying, due to the introduced water surface model.

#### 5.2.3. Wave Parameters

The wave parameters calculated with Method 1 and Method 2 differ, most seriously for the wavelength parameter. The differences are more pronounced at very long waves with small amplitudes (Series 3 and 4) and less evident at shorter waves or high amplitudes (Series 1, 2 and 5). In cases of small amplitude to wavelength ratios, the stability of the solution ought to be brought into discussion. Nonetheless, the subject has not been given further insight within this work.

## 6. Conclusions

Measurement of a difficult surface was approached with the collinearity equation, treating it as diffuse, and with the piecewise linear equations, when restituted solely from the specular reflections visible on its surface, but coming from a set of lamps hung from the ceiling. The accuracies obtained on physical targets floating on the surface, counted for the entire acquisition length, were more favorable for the latter method, falling between 1 and 3 mm.

The concept of using the lamps’ reflections for metrology was partially driven by the fact that lamps in ship testing facilities appear predominantly in the same configurations. They provide a calibration field at no labor cost, of high reliability, making the methodology universal and easy to re-apply. The diffuse method, on the contrary, necessitates extra work to establish a net of targets to be placed on the water. The points are then prone to sinking, partial submergence, occlusions or drift.

The superiority of the specular over the diffuse approach is in full-field *versus* single-point shape modeling. To install a net of points that spans a large area is infeasible; therefore, one is constrained to local observations. On the contrary, the number of specular highlights is a multiplication of every point in the calibration field by the number of cameras within the imaging system. Their allocation over the measurement volume is steerable by the camera placement. If, however, bound to using the diffuse approach and aiming at full-field data collection, eliciting the water shape over extended surfaces may be possible through the adoption of patches of nets in strategic areas. In both conditions, large field modeling demands very careful planning, especially for complex-shaped surfaces. The model definition must contain just enough parameters, whereas the observations ought to deliver enough data for their recovery.

A noteworthy aspect of the specular approach is the magnifying effect present on the water surface. Observed highlights undergo an apparent motion under the deforming surface shape. The motion magnitude and trajectory are known from the law of reflection. This depends on the camera to surface relation and the relation of the point at which reflection is observed (in its real position) to its reflection on the surface (virtual position). By modifying the distance between the reflecting surface and the calibration field (real points), the motion magnitude is changed proportionally. Put differently, very small surface deformations can render large highlight displacements for a sufficiently distant calibration field.

An important issue to consider when doing least squares adjustment, true for the diffuse and specular methods, is the initial approximations of all unknowns. Unless their values are known well enough, the success of the adjustment is put under question. At small amplitudes, the longer the wave is, the more precise must be the approximations. If approximations are imprecise, divergence or convergence to an incorrect solution is highly probable. Reliability measures output from the covariance matrices of the adjustment may serve to evaluate the credibility of the results. However, this has not been investigated within this work.

The model of the wave shape assumes single-frequency oscillations. In large field observations, this assumption is often violated, as has been observed in the presented work. If the assumption is violated and the model becomes insufficient to describe the phenomena, one may still: (i) use the simple model to observe the surface locally, or (ii) extend it to involve wave time-varying components, eventually modeling its shape with a wave being the sum of two elementary waves.

## Appendix A

(A1) $$\begin{array}{cc} f^{surf}=f_{0,i}^{surf}+\frac{\partial f^{surf}}{\partial A}dA+\frac{\partial f^{surf}}{\partial T}dT+\frac{\partial f^{surf}}{\partial \lambda }d\lambda +\frac{\partial f^{surf}}{\partial \phi }d\phi +\frac{\partial f^{surf}}{\partial y_{i}^{*}}dy_{i}^{*}+\frac{\partial f^{surf}}{\partial z_{i}^{*}}dz_{i}^{*} & =\\ f_{0,i}^{surf}+\frac{\partial f^{surf}}{\partial A}dA+\frac{\partial f^{surf}}{\partial T}dT+\frac{\partial f^{surf}}{\partial \lambda }d\lambda +\frac{\partial f^{surf}}{\partial \phi }d\phi +\\ \underset{\frac{\partial f^{surf}}{\partial X},\frac{\partial f^{surf}}{\partial Y},\frac{\partial f^{surf}}{\partial Z},\frac{\partial f^{surf}}{\partial \omega },\frac{\partial f^{surf}}{\partial \Phi },\frac{\partial f^{surf}}{\partial \kappa },\frac{\partial f^{surf}}{\partial T^{GCS\to LCS}}}{\underset{︸}{\sum_{K\in \{X_{i},Y_{i},Z_{i},\omega ,\Phi ,\kappa ,T^{GCS\to LCS}\}}\;\frac{\partial f^{surf}}{\partial y_{i}^{*}}\cdot \frac{\partial y_{i}^{*}}{\partial K}\;dK+\frac{\partial f^{surf}}{\partial z_{i}^{*}}\cdot \frac{\partial z_{i}^{*}}{\partial K}\;dK}} \end{array}$$

(A2) $$\begin{array}{cc} f^{refl}=f_{0,i}^{refl}+\frac{\partial f^{refl}}{\partial x_{O}^{*}}dx_{O}^{*}+\frac{\partial f^{refl}}{\partial y_{O}^{*}}dy_{O}^{*}+\frac{\partial f^{refl}}{\partial z_{O}^{*}}dz_{O}^{*}+\\ \frac{\partial f^{refl}}{\partial x_{R}^{*}}dx_{R}^{*}+\frac{\partial f^{refl}}{\partial y_{R}^{*}}dy_{R}^{*}+\frac{\partial f^{refl}}{\partial z_{R}^{*}}dz_{R}^{*}+\\ \frac{\partial f^{refl}}{\partial N_{x^{*},i}}dN_{x^{*},i}+\frac{\partial f^{refl}}{\partial N_{y^{*},i}}dN_{y^{*},i}+\frac{\partial f^{refl}}{\partial N_{z^{*},i}}dN_{z^{*},i} & =\\ f_{0,i}^{refl}+\sum_{j\in \{O,R\}}\;\sum_{K\in \{\omega ,\Phi ,\kappa ,T^{GCS\to LCS}\}}\frac{\partial f^{refl}}{\partial x_{j}^{*}}\cdot \frac{\partial x_{j}^{*}}{\partial K}dK+\frac{\partial f^{refl}}{\partial y_{j}^{*}}\cdot \frac{\partial y_{j}^{*}}{\partial K}dK+\frac{\partial f^{refl}}{\partial z_{j}^{*}}\cdot \frac{\partial z_{j}^{*}}{\partial K}dK+\\ \sum_{j\in \{x^{*},y^{*},z^{*}\}}\sum_{K\in \{X_{i},Y_{i},Z_{i},\omega ,\Phi ,\kappa ,T^{GCS\to LCS}\}}\;\frac{\partial f^{refl}}{\partial N_{j,i}}\cdot \frac{\partial N_{j,i}}{\partial K}\;dK \end{array}$$

(A3) $$\begin{array}{cc} f^{copl}=f_{0,i}^{copl}+\frac{\partial f^{copl}}{\partial x_{O}^{*}}dx_{O}^{*}+\frac{\partial f^{copl}}{\partial y_{O}^{*}}dy_{O}^{*}+\frac{\partial f^{copl}}{\partial z_{O}^{*}}dz_{O}^{*}+\\ \frac{\partial f^{copl}}{\partial x_{R}^{*}}dx_{R}^{*}+\frac{\partial f^{copl}}{\partial y_{R}^{*}}dy_{R}^{*}+\frac{\partial f^{copl}}{\partial z_{R}^{*}}dz_{R}^{*}+\\ \frac{\partial f^{copl}}{\partial N_{x^{*},i}}dN_{x^{*},i}+\frac{\partial f^{copl}}{\partial N_{y^{*},i}}dN_{y^{*},i}+\frac{\partial f^{copl}}{\partial N_{z^{*},i}}dN_{z^{*},i} & =\\ f_{0,i}^{copl}+\sum_{j\in \{O,R\}}\;\sum_{K\in \{\omega ,\Phi ,\kappa ,T^{GCS\to LCS}\}}\frac{\partial f^{copl}}{\partial x_{j}^{*}}\cdot \frac{\partial x_{j}^{*}}{\partial K}dK+\frac{\partial f^{copl}}{\partial y_{j}^{*}}\cdot \frac{\partial y_{j}^{*}}{\partial K}dK+\frac{\partial f^{copl}}{\partial z_{j}^{*}}\cdot \frac{\partial z_{j}^{*}}{\partial K}dK+\\ \sum_{j\in \{x^{*},y^{*},z^{*}\}}\sum_{K\in \{X_{i},Y_{i},Z_{i},\omega ,\Phi ,\kappa ,T^{GCS\to LCS}\}}\;\frac{\partial f^{copl}}{\partial N_{j,i}}\cdot \frac{\partial N_{j,i}}{\partial K}\;dK \end{array}$$

where:

(A4) $$\begin{array}{c} N_{{x^{*}}_{,i}}=\frac{\partial f^{surf}}{\partial y_{i}^{*}}\cdot \frac{\partial y_{i}^{*}}{\partial X_{i}}+\frac{\partial f^{surf}}{\partial z_{i}^{*}}\cdot \frac{\partial z_{i}^{*}}{\partial X_{i}}\\ N_{{y^{*}}_{,i}}=\frac{\partial f^{surf}}{\partial y_{i}^{*}}\cdot \frac{\partial y_{i}^{*}}{\partial Y_{i}}+\frac{\partial f^{surf}}{\partial z_{i}^{*}}\cdot \frac{\partial z_{i}^{*}}{\partial Y_{i}}\\ N_{{z^{*}}_{,i}}=\frac{\partial f^{surf}}{\partial y_{i}^{*}}\cdot \frac{\partial y_{i}^{*}}{\partial Z_{i}}+\frac{\partial f^{surf}}{\partial z_{i}^{*}}\cdot \frac{\partial z_{i}^{*}}{\partial Z_{i}} \end{array}$$
